# The first chromosomal-level genome assembly and annotation of white suckerfish *Remora albescens*

**DOI:** 10.1038/s41597-024-03363-4

**Published:** 2024-05-22

**Authors:** Chaowei Zhou, Qi Liu, Yinquan Qu, Ying Qiao, Tianxiang Gao, Danyang Wang

**Affiliations:** 1 College of Fisheries, Southwest University, Chongqing, 402460 China; 2 Integrative Science Center of Germplasm Creation in Western China (Chongqing) Science City, Southwest University, Chongqing, 402460 China; 3 Wuhan Onemore-tech Co., Ltd, Wuhan, 430000 China; 4 Fisheries College, Zhejiang Ocean University, Zhoushan, 316022 China; 5 Key Laboratory of Tropical Marine Ecosystem and Bioresource, Fourth Institute of Oceanography, Ministry of Natural Resources, Beihai, 536000 China; 6 MOE Key Laboratory of Marine Genetics and Breeding, College of Marine Life Sciences, Ocean University of China, Qingdao, 266100 China

**Keywords:** Agricultural genetics, Genome assembly algorithms

## Abstract

*Remora albescens*, also known as white suckerfish, recognized for its distinctive suction-cup attachment behavior and medicinal significance. In this study, we produced a high-quality chromosome-level genome assembly of *R. albescens* through the integration of 23.87 Gb PacBio long reads, 64.54 Gb T7 short reads, and 88.63 Gb Hi-C data. Initially, we constructed a contig-level genome assembly totaling 605.30 Mb with a contig N50 of 23.12 Mb. Subsequently, employing Hi-C technology, approximately 99.68% (603.38 Mb) of the contig-level genome was successfully assigned to 23 pseudo-chromosomes. Through the integration of homologous-based predictions, ab initio predictions, and RNA-sequencing methods, we successfully identified a comprehensive set of 22,445 protein-coding genes. Notably, 96.36% (21,629 genes) of these were effectively annotated with functional information. The genome assembly achieved an estimated completeness of 98.1% according to BUSCO analysis. This work promotes the applicability of the *R. albescens* genome, laying a solid foundation for future investigations into genomics, biology, and medicinal importance within this species.

## Background & Summary

**Fig. 1:**
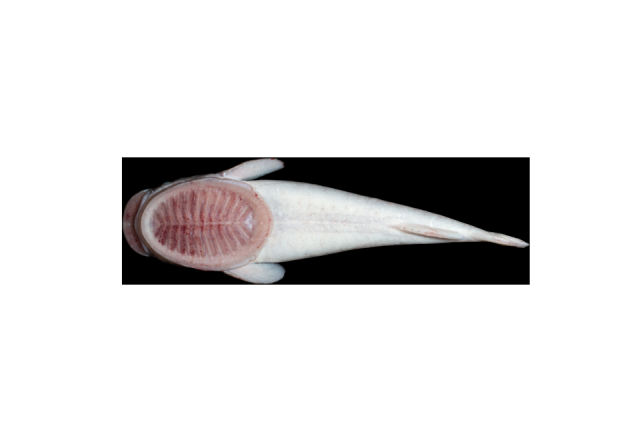
Morphological characteristics of *R. albescens*.

*Remora albescens*, namely white suckerfish or white remora, are in the Echeneidae family, order Carangiformes, and inhabit warm seas (Fig. 1). Similar to other members of the Echeneidae family, white suckerfish have evolved front dorsal fin sucking discs, which extend from the top of the head to the tips of their pectoral fins, consisting of 13-14 plates^1^. These adaptations enable them to adhere to smooth surfaces through suction, and they spend majority of their lives clinging to a host animal, such as a manta ray or a shark^2^. They frequently affix themselves to the body, as well as within the gill chamber and the mouth of the host^2^. The relationship between a white suckerfish and its host is typically considered a form of commensalism, specifically phoresy. Besides their unique biological characteristics, the white suckerfish are used in traditional Chinese medicine for their positive impact on lung and spleen-stomach health^3^, which grants them considerable medicinal value and commercial benefits.

High-quality reference genomes are instrumental in facilitating a deep understanding and comprehensive screening of the genetic foundation and variations linked to crucial traits. This knowledge allows us to gain insights into and effectively harness the biological characteristics of the species for various purposes. Currently, the genome of the white suckerfish has not been sequenced, impeding our exploration of genetic basis behind their biological features and behaviours. Overall, a high-quality chromosome-level reference genome will contribute to a profound comprehension of the genetic mechanisms responsible for the medicinal value of *R. albescens*.

In this study, through the integration of PacBio High fidelity (HiFi) long-reads, T7 paired-end sequencing short-reads and high-throughput chromatin capture (Hi-C) sequencing data (Table 1), we introduce the first chromosomal-level genome assembly of *R. albescens*. The assembly yielded a genome of 605.30 Mb, composed of 158 contigs, with a contig N50 length of 23.12 Mb. In total, 603.38 Mb, covering 99.68% of the contig-level genome, were accurately mapped onto 23 chromosomes by using Hi-C data. The BUSCO alignment analysis indicated that our ultimate assembly contained 3,571 (98.1%) complete BUSCOs. In conclusion, this high-quality chromosomal-level reference genome establishes a valuable foundation for comprehending the biological characteristics and conducting further research into the medicinal value of the *R. albescens*.

**Table 1:** Statistics of sequencing data for *Remora albescens* genome assembly and annotation.

Library type	Tissue	Raw data (Gb)	Clean data (Gb)	Average read length (bp)
BGISEQ T7	Muscle	66.21	64.54	150
PacBio HiFi	Muscle	429.05	23.87	18,233
Hi-C	Liver	88.75	88.63	150
RNA-seq	Pooled	6.12	6.01	150

**Table 2:** Comparison of the *R. albescens* genome assembly metrics with the *E. naucrates*.

	*R. albescens* (This study)	*E. naucrates*
Sequenced genome size (Mb)	605.30	544.2
Contig N50 (Mb)	23.12	12.4
Scaffold N50 (Mb)	25.66	23.3
Gap size (N’s per 100 kbp)	1.75	110.1

## Methods

### Fish sample collection and preparation

A single fish, measuring 18 centimeters in length, was obtained from Northern South China Sea in June 2022 (Fig. 1). The collection of the sampled fish for this study was conducted in accordance with the guidelines and regulations set forth by the Animal Care and Use Committee of Fisheries College of Zhejiang Ocean University, as indicated by Animal Ethics no. 1067. Tissues from the *R. albescens* were collected and preserved in liquid nitrogen until DNA or RNA extraction. Wherein, muscle and liver tissues were utilized for DNA sequencing to implement the genome assembly. Kidney, spleen, fin, gill and sucker tissues were utilized for RNA sequencing.

### WGS BGISEQ library and PacBio library construction, sequencing and contig-level assembly

According to the standard phenol/chloroform extraction instruction, the whole-genome sequencing (WGS) libraries were prepared by extracting genomic DNA from muscle tissues.

**Fig. 2:**
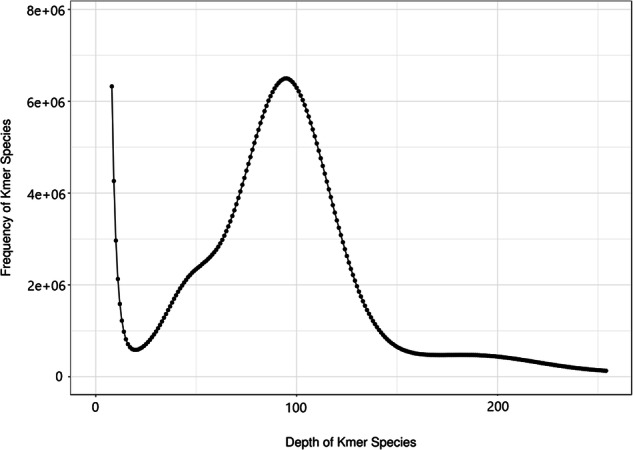
K-mer distribution of *R. albescens*.

To obtain BGISEQ short reads, the DNA sample underwent evaluation through 1% agarose gel electrophoresis and the Pultton DNA/Protein Analyzer (Plextech). Subsequently, a paired-end library with an insert size of 300 bp to 350 bp was constructed following the BGISEQ standard protocol. Afterward, the DNA sample was purified, quantified, and subjected to sequencing from both ends using the BGISEQ-T7 sequencing platform. BGISEQ sequencing resulted in a total of 66.21 Gb raw reads (Table 1). Following a filtering process utilizing fastp v0.23.2^4^ with default parameters, which aimed to eliminate low-quality, short reads, adapters and redundant sequences, a total of 64.54 Gb clean reads were obtained (Table 1). Then by using GCE v1.0.0 software^5^, *K*-mer analysis was performed to estimate the genome size and heterozygosity for *R. albescens*, which were 563 Mb and 0.63%, respectively (Fig. 2).

To obtain PacBio long reads, the DNA sample was first evaluated using Nanodrop, Qubit and agarose gel electrophoresis. Then, the library with a fragment size of 20 kb was created utilizing the SMRTBell template preparation kit 1.0 following the manufacturer’s instructions. Afterward, the DNA sample was subjected to sequencing using the PacBio Sequel II platform in Circular Consensus Sequence (CCS) mode. After removing low-quality sequences using the CCS v6.0.0 algorithm with default parameters, a sum of 23.87 Gb high-precision reads with an N50 value of 18.88 kb were obtained. With these HiFi reads, the initial contigs were assembled using the Hifiasm v0.16.1^6^ and the purge_haplotigs algorithms^7^ with the default settings. The assembly yielded a 605.30 Mb genome with a maximum contig size of 51.46 Mb.

### Hi-C library preparation, sequencing and chromosomal-level assembly

**Fig. 3:**
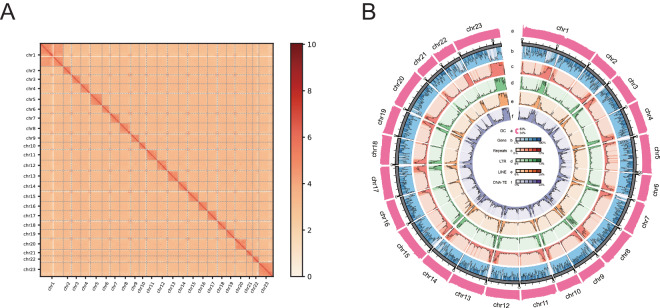
Genome assembly of *R. albescens*. (**A**) Hi-C interaction matrix for *R. albescens*. (**B**) Circos plot from outer to inner layers depicts the following: (a) GC content; (b) gene density; (c) repeat density; (d) LTR retroelement density; (e) LINE density; and (f) DNA transposons density. a-f were drawn in 500-kb sliding windows.

The contigs obtained in the previous step were anchored onto chromosomes using Hi-C data. In a nutshell, 1 g of liver tissue from *R. albescens* was treated with 1% formaldehyde for 20 minutes at 20–25 °C temperature to facilitate the coagulation of proteins implicated in chromatin interactions. Next, DNA was digested using MboI and the overhangs of the resulting restriction fragments were labeled with biotinylated nucleotides, after which they were ligated within a confined volume. Following the cross-link reversal, the ligated DNA was purified and fragmented to a size range of 300–500 bp. Following this step, ligation junctions were extracted by streptavidin beads and subjected to sequencing from both ends using the BGISEQ-T7 sequencing platform, producing a total of 88.75 Gb raw data (Table 1). After removing low-quality sequences and adapters, and only retaining paired-end reads, both of which are longer than 50 bp, with fastp v0.23.2^4^ software, a sum of 88.63 Gb clean data were acquired (Table 1). We utilized the HiCUP pipeline^8^ to obtain credible and nonredundant contigs interaction matrix, and then anchored the contigs onto chromosomes by using 3D-DNA pipeline^9^. Juicebox Assembly Tools^10^ was utilized for manual error correction to rectify any occurrences of chromosome inversion and translocation. Finally, 603.38 Mb (~99.63%) of contig-level assembled sequences were positioned onto 23 pseudo-chromosomes (Fig. 3A).

### RNA library construction and sequencing

Total RNA was extracted from the five tissues, including kidney, spleen, fin, gill and sucker, of the *R. albescens* using TRIzol reagent (Invitrogen). To evaluate RNA quality, we utilized the NanoDrop ND-1000 spectrophotometer (Labtech) and the 2100 Bioanalyzer (Agilent Technologies). The paired-end reads were sequenced using the BGISEQ-T7 Platform. Overall, 6.01 Gb of clean data were obtained following filtering process utilizing fastp v0.23.2^4^ with default settings to eliminate low-quality and short reads, as well as trim adapters and polyG tails (Table 1).

### Repetitive elements annotation

Repeat elements in the *R. albescens* genome were systematically identified using a dual approach, incorporating both homology-based searches and ab initio predictions. The ab initio prediction of repeat elements was carried out through two tools, namely Tandem Repeat Finder v4.09^11^ and LTR_FINDER_parallel v1.1^11^ with default parameters. Subsequently, newly discovered repeats were predicted using RepeatMasker v4.0.9^12^, based on the de novo repetitive sequence library that was constructed using LTR_FINDER_parallel and RepeatModeler v2.0^13^, RepeatMasker v4.0.9 and RepeatProteinMask v4.1.0 (http://www.repeatmasker.org) were used to identify known repeat elements with the Repbase v20181026 database^14^. In total, 18.04% of the *R. albescens* genome were identified as repetitive sequences (Fig. 3B). Among these repeat elements, DNAs, LTRs, LINEs, and SINEs constituted 6.98%, 2.49%, 5.41%, and 1.69% of the genome, respectively (Table 3).

**Table 3:** Statistics on transposable elements in the *R. albescens* genome.

Type	Repbase TEs		TE protiens		De novo		Combined TEs	
	Length (Bp)	% in genome	Length (Bp)	% in genome	Length (Bp)	% in genome	Length (Bp)	% in genome
DNA	28,650,770	4.69	5,755,996	0.94	23,633,757	3.87	42,590,519	6.98
LINE	25,131,255	4.12	18,298,621	3	18,217,820	2.98	33,034,151	5.41
SINE	6,147,830	1.01	0	0	4,372,739	0.72	10,348,445	1.69
LTR	10,344,266	1.69	5,754,470	0.94	8,481,267	1.39	15,182,686	2.49
Satellite	1,391,046	0.23	0	0	1,010,060	0.17	2,210,026	0.36
Simple_repeat	0	0	0	0	0	0	0	0
Other	2,147	0	363	0	0	0	2,510	0
Unknown	294,857	0.05	13,479	0	14,067,211	2.3	14,335,996	2.35
Total	67,550,139	11.06	29,804,936	4.88	68,459,076	11.21	110,137,154	18.04

### Gene prediction and annotation

Utilizing the repeat-masked genome as a basis, three strategies, comprising ab initio prediction, homologous prediction and RNA-sequencing method, were employed to predict protein-coding genes within the *R. albescens* genome. Ab initio prediction was conducted utilizing Augustus v3.3.2^15^ and Genscan^16^ software. Simultaneously, homologous prediction relied on protein sequences from various annotated species, comprising *Seriola lalandi* (RefSeq assembly accession: GCF_002814215.2), *Seriola dumerili* (RefSeq assembly accession: GCF_002260705.1), *Echeneis naucrates* (RefSeq assembly accession: GCF_900963305.1), *Takifugu rubripes* (RefSeq assembly accession: GCF_901000725.2), *Gasterosteus aculeatus* (RefSeq assembly accession: GCF_016920845.1), and *Danio rerio* (RefSeq assembly accession: GCF_000002035.6). The protein sequences above were retrieved from the NCBI database and then aligned with the *R. albescens* genome utilizing tblastn tool (e-value ≤ 1e-5). Subsequently, the homologous sequences were aligned with the corresponding proteins with Genewise v2.4.0^17^ to predict detailed gene structures. The RNA-seq dataset were aligned to the assembled genome by using HISAT2 v2.1.0^18^ with default settings, and the predicted transcripts were identified by using StringTie v1.3.5^19^ and TransDecoder v5.1.0 (https://github.com/TransDecoder/TransDecoder) with default settings. Three gene model predictions were merged using MAKER v2.31.10^20^. Based on that, we further refined the gene set using HiFAP (Wuhan OneMore Tech Co., Ltd., https://www.onemore-tech.com/) with high-quality transcripts and homology annotation results, resulting in a final gene set with a total number of protein-coding genes of 22,445 genes (Fig. 3B and Table 4).

**Table 4:** Statistics of gene predictions in the *R. albescens* genome.

	Gene set	Number	Average gene length (bp)	Average CDS length (bp)	Average exon per gene	Average exon length (bp)	Average intron length (bp)
denovo	Genscan	27,676	14,990.17	1,625.82	9.13	178.05	1,643.61
	AUGUSTUS	25,679	10,215.07	1,432.42	8.13	176.20	1,231.86
Homolog	Seriola_lalandi	30,983	10,793.53	1,515.28	8.60	176.25	1,221.27
	Seriola_dumerili	28,457	11,143.41	1,558.54	8.95	174.18	1,206.00
	Echeneis_naucrates	28,147	11,530.16	1,610.79	9.22	174.73	1,206.95
	Takifugu_rubripes	27,988	10,957.90	1,525.02	8.77	173.92	1,214.26
	Gasterosteus_aculeatus	29,609	10,829.01	1,530.11	8.67	176.58	1,213.10
	Danio_rerio	30,619	10,997.90	1,414.55	8.06	175.40	1,356.47
RNAseq	Trans.orf	15,624	19,722.53	1,962.73	12.79	361.56	1,280.74
BUSCO		3,648	9,995.01	1,814.18	11.85	153.05	753.76
MAKER		21,220	16,829.75	1,786.02	11.05	309.27	1,333.88
HiCESAP		22,445	16,428.03	1,754.98	11.10	327.35	1,266.46

The functional annotation of the predicted protein-coding gene sets was performed using BLASTp (e-value ≤ 1e-5) with the diamond v2.0.8 software^21^ based on six databases, including Swiss-Prot v2023-03-01^22^, NCBI nonredundant protein (NR) v2023-04-01, Kyoto Encyclopedia of Genes and Genomes (KEGG) v2023-01-01 (http://www.genome.jp/kegg/), TrEMBL v2023-03-01 (http://www.uniprot.org), eukaryotic orthologous groups of proteins (KOG) v2003-03-01^23^ and AnimalTFDB v4.0 (http://bioinfo.life.hust.edu.cn/AnimalTFDB4/?#/). Additionally, protein structural domain predictions of gene sets were performed based on InterPro and Pfam databases utilizing InterProScan v5.61-93.0^24^ with parameters “–goterms–pathways -dp”. As a result, 96.36% (21,629 genes) of the total predicted genes were successfully annotated. (Table 5).

**Table 5:** Summary of functional annotations for predicted genes of the *R. albescens* genome.

		Number	Percent (%)
Total		22,445	
Annotated	Merged	21,629	96.36
	InterPro	19,833	88.36
	GO	15,145	67.48
	KEGG_ALL	21,308	94.93
	KEGG_KO	13,853	61.72
	Swissprot	19,221	85.64
	TrEMBL	21,450	95.57
	TF	3,440	15.33
	Pfam	19,177	85.44
	NR	21,585	96.17
	KOG	17,819	79.39
Unannotated		816	3.64

### Non-coding RNA prediction and annotation

According to the miRBase^25^ and rfam^26^ databases, the microRNAs (miRNAs), ribosomal RNAs (rRNAs) and small nuclear RNAs (snRNAs) were annotated utilizing INFERNAL v1.1^27^. The transfer RNAs (tRNAs) were predicted by using tRNAscan-SE v1.3.1^28^. Consequently, 829 miRNAs, 1,832 rRNAs, 820 snRNAs and 7,033 tRNAs were predicted within the *R. albescens* genome (Table 6).

**Table 6:** Statistics of ncRNA in the *R. albescens* genome.

Type		Copy	Average length(bp)	Total length(bp)	% of genome
miRNA		829	86	71,642	0.01
tRNA		7,033	76	535,999	0.09
rRNA	rRNA	1,832	313	573,150	0.09
	18 S	209	1,788	373,642	0.06
	28 S	0	0	0	0.00
	5.8 S	207	154	31,878	0.01
	5 S	1,416	118	167,630	0.03
snRNA	snRNA	820	147	120,865	0.02
	CD-box	128	109	13,967	0.00
	HACA-box	66	151	9,940	0.00
	splicing	620	154	95,541	0.02
	scaRNA	6	236	1,417	0.00

## Data Records

The raw sequencing data for *R. albescens* in this study is available from the Sequence Read Archive (SRA) under Bioproject number PRJNA1036795, which includes WGS T7 sequencing data (SRR26831100^29^), Pacbio HiFi sequencing data (SRR26831099^30^), Hi-C sequencing data (SRR26831098^31^), and RNA sequencing data (SRR28537587^32^). The assembled genome of *R. albescens* has been deposited in GenBank under accession JAXCVL000000000^33^. Additionally, files contained the assembled genome, protein-coding gene annotation, non-coding RNA prediction, and repeat annotation of *R. albescens* have been made available in the Figshare database^34^.

## Technical Validation

Our initial assessment of the continuity of the *R. albescens* genome assembly was conducted using QUAST v5.2.0^35^. The contig N50 reaches 23.12 Mb and the genome displays a minimal number of gaps (1.75 per 100 kbp), which exhibits better assembly performance than closely related species (*Echeneis naucrates*: GCA_900963305.1) (Table 2). Next, we remapped T7 clean short reads and PacBio clean long reads to the *R. albescens* genome using BWA^36^ and Minimap2^37^, yielding mapping rates of 99.83%, 99.96% and coverage rates (at least 4X) of 99.61%, 99.76%, respectively (Table 7). Furthermore, the completeness of the *R. albescens* genome was evaluated using Benchmarking Universal Single-Copy Orthologs (BUSCO, v5.1.0)^38^ with the actinopterygii_odb10 database. The analysis revealed that the genome assembly contained 3,571 (98.1%) complete BUSCO genes, comprising 3,551 (97.55%) single-copy BUSCO genes, 20 (0.55%) duplicated BUSCO genes, and 11 (0.3%) fragmented BUSCO genes (Table 8). Collectively, the comprehensive assessment indicates that the *R. albescens* genome serves as a high-quality reference genome.

**Table 7:** Statistics of T7 and PacBio data remapped to the *R. albescens* genome.

Sample name	Mapping rate(%)	Average sequencing depth	Coverage(%)	Coverage at least 4X(%)	Coverage at least 10X(%)	Coverage at least 20X(%)
**T7**	99.83	102.7	99.85	99.61	99.18	98.57
**PacBio**	99.96	38.83	99.99	99.76	99.17	95.71

**Table 8:** Statistics of BUSCO assessment in the *R. albescens* genome.

Type	BUSCOs number	Percentage(%)
Complete BUSCOs	3,571	98.1
Complete and single-copy BUSCOs	3,551	97.55
Complete and duplicated BUSCOs	20	0.55
Fragmented BUSCOs	11	0.3
Missing BUSCOs	58	1.59
Total BUSCO groups searched	3,640	100

## Data Availability

No specific custom codes were developed in this study. Data analyses were conducted following the guidelines and protocols provided by the developers of the respective bioinformatics tools, as detailed in the methods section.
